# The absolute and relative concentration-response patterns of air pollutants and the risk of Alzheimer’s disease

**DOI:** 10.1093/geroni/igaf122.249

**Published:** 2025-12-31

**Authors:** Igor Akushevich, Arseniy Yashkin, Larry Tupler, Meishuo Ouyang, Julia Kravchenko

**Affiliations:** Duke University, Durham, North Carolina, United States; Duke University, Durham, North Carolina, United States; Duke University, Durham, North Carolina, United States; Duke University School of Medicine, Durham, North Carolina, United States; Duke University School of Medicine, Durham, North Carolina, United States

## Abstract

Existing literature lacks information on the relationship between exposure to ambient air contaminants and Alzheimer’s disease (AD) risk represented by simultaneous assessment of absolute and relative concentration-response patterns. This study focuses on quantifying PM2.5-exposure and AD-risk relationships with detailed analyses of the role of methodologic limitations and sources of bias (e.g., population heterogeneity) and identifying notable subpopulation differences and populations most vulnerable to PM2.5 exposure. Using Medicare and SEDAC data, we evaluated the effects of PM2.5 exposure on AD risk stratified by sex, race/ethnicity, and exposure level using absolute concentration-response functions based on age-adjusted rates and relative functions estimated via Cox models. The results show that chronic low-level exposures to PM2.5 were associated with higher AD risk which varied substantially across sex- and race-specific population groups. Females showed higher absolute incidence, while males exhibited steeper relative risk increases with rising PM2.5 concentrations. Native Americans, residing primarily in low-exposure areas, demonstrated the steepest rise in hazard with increasing PM2.5 concentrations; Black and Asian subpopulations exhibited the lowest relative ratios. Nonlinearities were detected in exposure-response relationships. AD risk in Hispanics was subject to notable geographic heterogeneity. This study provides a foundation for assessing the health impacts of recent U.S. air quality standards and their effects on AD risk in vulnerable populations. Future research should incorporate flexible modeling techniques (e.g., spline-based or piecewise methods) and integrate mechanistic studies to clarify the biological and social pathways linking PM2.5 to AD, explain observed disparities, and identify underlying causal factors.

